# OCTA and microperimetry in Behçet’s retinal vasculitis: subgroup analysis, structure–function correlation, and 12-month follow-up evaluation

**DOI:** 10.1186/s40942-026-00866-7

**Published:** 2026-06-26

**Authors:** Bruno F. A. Ferreira, Alex H. Higashi, Leandro L. Prado, Célio R. Gonçalves, Maria A. O. Haddad, Leandro C. Zacharias, Cleide G. Machado, Carlos E. Hirata, Joyce H. Yamamoto

**Affiliations:** 1 Department of Ophthalmology, Faculdade de Medicina, Hospital das Clinicas HCFMUSP, Universidade de Sao Paulo, 255 Dr. Enéas Carvalho de Aguiar Avenue, Sao Paulo, SP 05403900 Brazil; 2 Rheumatology Division, Faculdade de Medicina, Hospital das Clinicas HCFMUSP, Universidade de Sao Paulo, Sao Paulo, Brazil

**Keywords:** Behçet’s syndrome, Microperimetry, Optical coherence tomography angiography, Retinal microvasculature

## Abstract

**Background:**

In Behçet’s syndrome (BS)–associated retinal vasculitis, optical coherence tomography (OCT) angiography (OCTA) and microperimetry allow noninvasive assessment of retinal microvascular integrity and macular function, but integrated structure–function analyses across all retinal vascular plexuses during inactive disease remain limited. This study evaluated age- and sex-related differences, structure–function associations, and 12-month changes in inactive retinal vasculitis.

**Methods:**

This observational study included 14 patients (23 eyes; mean age 40.6 ± 11.7 years) with inactive BS-associated retinal vasculitis, followed every 4 months for 12 months. Retinal thickness, retinal nerve fiber layer and ganglion cell layer thickness on OCT, foveal avascular zone (FAZ) area and vascular density (VD) on OCTA, and macular sensitivity (MS) on microperimetry were analyzed. Exploratory subgroup analyses were performed according to sex, age, and qualitative OCTA findings. Associations were assessed using generalized estimating equations, and correlations were evaluated using Spearman’s coefficient.

**Results:**

Male sex, age < 45 years, and qualitative OCTA findings (perifoveal arcade disruption, non-perfusion areas, and microvascular abnormalities in any vascular plexus) were associated with more pronounced quantitative changes on OCT and OCTA (*P* < 0.05). VD showed strong inverse and direct correlations (*r* > 0.7) with FAZ area and retinal thickness, respectively, which should be interpreted with caution given the limited sample size. In the superficial vascular plexus (SVP), superior sector VD correlated directly with central MS (*r* = 0.722; *P* < 0.001). Structural and functional biomarkers remained stable throughout the 12-month follow-up in patients under clinical remission.

**Conclusions:**

Male sex and younger age appeared to be associated with patterns suggestive of greater macular structural and functional involvement in BS-associated retinal vasculitis. Qualitative OCTA findings, particularly involving the SVP and intermediate capillary plexus, were also associated with more pronounced structural and functional alterations. However, given the exploratory nature of the analyses and the limited sample size, these findings should be considered preliminary and warrant further investigation in larger, controlled studies.

## Introduction

Behçet’s syndrome (BS), or Behçet’s disease—a systemic vasculitis of unknown etiology characterized by periods of recurrence and remission—is diagnosed based on clinical findings [1]. Ocular involvement in 28%–70% of the patients is characterized by recurrent non-granulomatous panuveitis with retinal vasculitis and can cause irreversible vision loss if left untreated [2]. Visual prognosis depends on control or prevention of ocular reactivations [3, 4].

While fluorescein angiography (FA) is the standard exam for evaluating retinal vasculitis, it has limitations [5, 6]. It is an invasive test and does not allow visualization of all the microvascular plexuses of the retina [7]. In contrast, optical coherence tomography angiography (OCTA), a dye-free imaging method, provides a three-dimensional map of the retinal and choroidal microvasculature and enables a detailed evaluation of macular microvascular changes [8, 9].

Our recent study compared patients with inactive Behçet’s uveitis (BU), non-ocular BS, and healthy subjects [10]. The findings were significant and revealed that there was a thinning in the full retinal thickness and ganglion cell layer (GCL), as well as a reduction in the vascular density (VD) of the deep capillary plexus (DCP), particularly in the nasal sector. No significant difference was observed in the retinal nerve fiber layer (RNFL). Notably, a correlation between these microvascular changes and retinal sensitivity measured by microperimetry (MP) was observed in 30.4% of the eyes.

The current study aims to assess the superficial vascular plexus (SVP), the intermediate capillary plexus (ICP), and the DCP and to evaluate associations between OCTA findings and OCT, MP, and clinical parameters in patients with inactive BU. Furthermore, it monitored the retinal microvasculature and sensitivity in the macular area during a 12-month follow-up.

## Methods

### Study design and population

This observational study included 23 eyes of 14 patients (mean age: 40.6 ± 11.7 years) with BS-associated retinal vasculitis followed up between October 2018 and December 2020 at the Ophthalmology and Rheumatology Division of Hospital das Clinicas HCFMUSP, Faculdade de Medicina, Universidade de Sao Paulo, Sao Paulo, Brazil. The inclusion criteria were a BS diagnosis based on the International Criteria for Behçet’s Disease [11], age > 18 years, and inactive retinal vasculitis (based on clinical and FA) for at least 3 months. This study was approved by the Institutional Research Ethics Committee (protocol number 18393419.1.0000.0068), and written informed consent was obtained from all participants. All procedures adhered to the tenets of the Declaration of Helsinki and to the institution’s ethical guidelines and regulations. The exclusion criteria were isolated anterior uveitis, refractive error greater than 6.0 diopters, media opacity, retinal or optic disk diseases not related to BS (diabetic retinopathy, macular degeneration, or glaucoma), intraocular surgery < 6 months, and systemic diseases or medications that could affect the macula. The patients included in this study belong to the same cohort previously reported, in which comparisons with healthy subjects and patients with non-ocular Behçet’s syndrome were performed [10]. Therefore, the present study was designed as a focused extension to explore subgroup analyses and longitudinal changes in patients with inactive Behçet’s uveitis within this cohort.

Clinical data were obtained by reviewing the medical records and anamnesis during patient inclusion. The following data were collected: comorbidities, disease duration based on the first symptom, number of disease relapses, period of inactive disease, and treatment. Inflammatory activity in Behçet’s syndrome was assessed using the Brazilian-adapted Behçet’s Disease Current Activity Form (BR-BDCAF), with a score above five indicating active disease [12].

### Functional tests and exams

Best-corrected visual acuity (BCVA) and contrast sensitivity were measured using the Snellen chart at 4 m and the Pelli-Robson contrast sensitivity chart at 1 m, respectively. Measurements were converted to logMAR.

Further, FA (Spectralis^®^, Heidelberg Engineering, Heidelberg, Germany) was performed after intravenous injection of 2 mL of 20% sodium fluorescein, and angiograms in the early and late phases were recorded. The number of quadrants with vascular filling defects or vascular leakage in the peripheral retina was assessed. Quadrants were defined by horizontal and vertical lines at the fovea, with the peripheral retina excluding the central zone (macula, optic disc, and temporal vascular arcades) [13].

Macular sensitivity was studied using the MP-3 microperimeter (Nidek, Tokyo, Japan) in a dark room, with the contralateral eye occluded. All eyes were evaluated by the same examiner 30 min after the instillation of 1% tropicamide eye drops. Patients were allowed to adapt to mesopic conditions prior to testing. A 2° diameter red circle fixation target and a customized stimulus pattern, which consisted of a central point (foveolar) and 16 points (foveal and parafoveal) arranged in concentric rings with diameters of 4° and 8°, divided into four sectors, were used. Sensitivity measurements were obtained using a 4-2-1 scaling strategy, with a Goldmann III stimulus on a white background, a duration of 200 ms, a maximum luminance of 10,000 asb, and a dynamic range of 0–34 dB. Fixation stability was monitored using the device’s eye-tracking system, and only examinations with stable or relatively stable fixation were included. Tests with poor patient cooperation or significant tracking loss were excluded. The central point threshold was defined as central macular sensitivity (MS), while the average threshold of the 16 concentric points represented the mean MS. The same points were stimulated for comparison during follow-up using the MP-3 eye-tracking system. For the analysis, 30.0 ± 0.7 dB was considered the reference value for MS, and a variation of ± 4 dB between exams was tolerated [14].

### Structural exams

The OCT images were acquired with Spectralis OCT2 (Heidelberg Engineering, Heidelberg, Germany), with a minimum signal strength index value of 20 and 24-line radial scans. The Spectralis OCT2 software (version 6.9) automatically defined the segmentation and thickness of the mean foveal and parafoveal retina, RNFL, and GCL. Using enhanced depth imaging OCT, the subfoveal choroidal thickness was manually measured twice by the same examiner (B.F.A.F.) to ensure intraobserver consistency, and its average was used in the analysis.

OCTA scans over an area 3 × 3 mm centered on the fovea were also acquired with a Spectralis OCT2 (Heidelberg Engineering, Heidelberg, Germany). OCT angiograms with signal strength index values < 40 (due to artifacts, media opacities, blinking, or fixation loss) were excluded. The intraretinal layers were automatically segmented by the Spectralis OCT2 software (version 6.9) and were manually adjusted when necessary. *En face* images were generated for the SVP (from the RNFL to the inner plexiform layer), the ICP (from the outer 20% of the GCL to the mid-inner nuclear layer), and the DCP (from the mid-inner nuclear layer to the outer plexiform layer) [15, 16]. The same examiner (B.F.A.F.) outlined the (FAZ) in the full retina slab on two separate occasions to ensure intraobserver consistency, and its area and circularity (4π × area/perimeter^2^) were automatically calculated using ImageJ (National Institutes of Health, Bethesda, MD, USA; available at https://imagej.nih.gov/ij/download.html). To measure the VD, a square of 450 × 450 pixels was extracted from the original angiogram, converted into 8-bit files, and analyzed with ImageJ. The FAZ area was excluded from the image, which was binarized according to Otsu’s method and post-processed into the superior, inferior, temporal, and nasal sectors. Finally, the capillary VD was calculated for each sector using the following formula: VD (%) = white pixels/(total pixels – FAZ area) × 100. All quantitative variables were correlated. Qualitative findings included non-perfusion areas (NPA) in OCTA, seen as hypointense regions with absent or reduced capillary flow, and microvascular abnormalities characterized by dilatation (enlarged capillaries), tortuosity (increased vessel curvature), and rarefaction (widened intercapillary spaces).

### Statistical analysis

Given the rarity of Behçet’s syndrome–associated retinal vasculitis, both eyes were included when eligible in order to maximize the available data. To account for inter-eye correlation, generalized estimating equations (GEE) were applied, which are appropriate for clustered ophthalmic data and have been widely used in retinal imaging studies. Spearman’s correlations were calculated among all the quantitative biomarkers, and retinal plexus parameters were described according to qualitative parameters. The values for the categories of qualitative characteristics were compared using GEE with normal distribution and an identity link function, assuming an exchangeable correlation structure between the eyes of the same patient. Subgroup analyses, including sex- and age-based comparisons, were predefined as exploratory and hypothesis-generating rather than confirmatory. Cutoff points between 40 and 55 years were considered to analyze subgroups by age, and 45 years was determined as the most suitable cutoff point in our sample [17, 18]. The analyses were performed using IBM-SPSS software for Windows version 20.0 with a significance level of 5%. Due to the exploratory nature of this study and the rarity of Behçet’s syndrome–associated retinal vasculitis, a formal sample size or power calculation was not performed.

## Results

### Demographic and clinical data

All patients presented inactive systemic disease, with a mean (± SD) BR-BDCAF score of 1.14 ± 1.79. During the course of the disease, the systemic manifestations were oral ulceration (13 patients, 92.9%), genital ulceration (9 patients, 64.3%), skin-mucosa lesions (8 patients, 57.1%), arthritis (6 patients, 42.9%), vascular manifestation (1 patient, 7.1%) and neurological manifestation (1 patient, 7.1%). At inclusion, five eyes were excluded due to poor exam quality. Four of these eyes (80%) belonged to the younger subgroup (< 45 years), while only one belonged to the older subgroup (≥ 45 years). The BCVA was better than 20/50 (logMAR 0.4) in 21 eyes (91.3%), with a median of 20/20 (logMAR 0, range 0–0.7). The median of the contrast sensitivity was 1.35 (range 0.9–1.65). Twenty-one eyes (91.3%) had panuveitis, and two had posterior uveitis. The mean (± SD) total immunosuppressive drugs per patient was 3.5 ± 1.51. The mean (± SD) disease duration was 12.2 ± 6.2 years.

## Sex and age analysis

The analysis by sex (Table 1) demonstrated that male (*n* = 7, 12 eyes) differed (*P* < 0.05) from female patients (*n* = 7, 11 eyes), with greater thinning in retinal thickness (superior, nasal, and inferior sectors), RNFL (inferior and nasal sectors), GCL (nasal sector), and RNFL + GCL complex (nasal, temporal, and inferior sectors). Significant differences were also observed in the sensitivity on MP (nasal and inferior sectors) and VD on OCTA in SVP (nasal, temporal, and inferior sectors) and ICP (nasal, temporal, and inferior sectors).

**Table 1:** Sex-related differences in OCT, OCTA, and microperimetry parameters in Behçet’s retinal vasculitis

Variable*	Female (11 eyes)	Male (12 eyes)	*P*†
OCT—Thickness (µm)			
Full retina			
Superior	333.6 ± 22.1 | 334 (307; 371)	308.3 ± 44.5 | 322.5 (198; 370)	0.028
Nasal	318.4 ± 35.5 | 317 (263; 369)	280.2 ± 40.4 | 294 (222; 332)	0.021
Inferior	326.7 ± 24.1 | 318 (283; 354)	296.9 ± 52.6 | 316.5 (173; 356)	0.049
RNFL			
Nasal	20.9 ± 3.4 | 20 (15; 26)	17.5 ± 2.7 | 16.5 (14; 23)	0.007
Inferior	26.6 ± 4.2 | 26 (17; 32)	21.4 ± 6.8 | 23.5 (7; 29)	0.010
CGL			
Inferior	45.8 ± 13 | 45 (25; 66)	33.5 ± 11.2 | 33 (18; 50)	0.017
RNFL + GCL Complex			
Nasal	66.7 ± 15.5 | 65 (43; 92)	51 ± 12.7 | 51 (32; 68)	0.006
Temporal	64.1 ± 9.8 | 66 (40; 77)	53.8 ± 15.2 | 60 (21; 70)	0.047
Inferior	75.3 ± 11.7 | 75 (54; 96)	61.4 ± 20.1 | 66.5 (15; 79)	0.042
OCTA—Vascular density			
SVP			
Global	0.249 ± 0.013 | 0.248 (0.224; 0.271)	0.214 ± 0.028 | 0.211 (0.17; 0.261)	< 0.001
Nasal	0.258 ± 0.026 | 0.261 (0.22; 0.31)	0.197 ± 0.036 | 0.194 (0.133; 0.246)	< 0.001
Temporal	0.248 ± 0.021 | 0.243 (0.219; 0.285)	0.209 ± 0.039 | 0.21 (0.139; 0.266)	< 0.001
Inferior	0.244 ± 0.015 | 0.244 (0.226; 0.268)	0.216 ± 0.037 | 0.215 (0.158; 0.278)	0.017
ICP			
Global	0.305 ± 0.02 | 0.3 (0.267; 0.344)	0.267 ± 0.04 | 0.273 (0.187; 0.323)	< 0.001
Nasal	0.303 ± 0.029 | 0.31 (0.251; 0.342)	0.26 ± 0.048 | 0.266 (0.165; 0.322)	< 0.001
Temporal	0.313 ± 0.025 | 0.318 (0.273; 0.367)	0.271 ± 0.046 | 0.271 (0.185; 0.33)	0.002
Inferior	0.302 ± 0.021 | 0.304 (0.277; 0.335)	0.257 ± 0.05 | 0.267 (0.175; 0.339)	< 0.001
MP—Macular sensitivity (dB)			
Nasal	23.9 ± 4.3 | 23.3 (16.8; 31.2)	14.6 ± 11 | 14.8 (0; 31)	0.012
Inferior	26.8 ± 4 | 28.2 (18.5; 30.8)	18.5 ± 12.7 | 24.1 (0; 32)	0.035

GCL, ganglion cell layer; ICP, intermediate capillary plexus; MP, microperimetry; OCTA, optical coherence tomography angiography; OCT, optical coherence tomography; RNFL, retinal nerve fiber layer; SVP, superficial vascular plexus

*Represented by mean ± SD and median (min.; max.), respectively

†Generalized estimation equations with normal distribution and identity link function with exchangeable correlation matrix between eyes

Regarding the age of the patients (Table 2) at inclusion in the study, eight patients (12 eyes) were aged below 45 years (mean ± SD = 32.6 ± 7.6; median = 31.5, range 18–43), and six patients (11 eyes) were aged 45 and over (mean ± SD = 51.3 ± 5.9; median = 51, range 45–62). Within the subgroup aged below 45 years, 3 patients were female (3 eyes) and 5 were male (9 eyes). In the subgroup aged 45 years and over, 4 were female (8 eyes) and 2 were male (3 eyes). Younger patients had reductions (*P* < 0.05) in the VD in SVP (superior sector), ICP (global, superior, nasal, and inferior sectors), and DCP (superior sector). The mean age at disease diagnosis (*P* = 0.023) and the disease duration (*P* = 0.019) in patients younger than 45 years were 23.6 ± 8.9 and 9 ± 5.2 years, respectively, while those in patients aged 45 years and over were 34.8 ± 6.6 and 16.5 ± 5.1 years.

**Table 2:** Age-related differences in OCT, OCTA, and microperimetry parameters in Behçet’s retinal vasculitis

Variable*	< 45 years (*n* = 12 eyes)	≥ 45 years (11 eyes)	*P*†
OCTA—Vascular density			
SVP			
Superior	0.228 ± 0.028 | 0.218 (0.18; 0.278)	0.254 ± 0.024 | 0.255 (0.191; 0.28)	0.022
ICP			
Global	0.269 ± 0.036 | 0.276 (0.187; 0.322)	0.302 ± 0.031 | 0.314 (0.223; 0.344)	0.004
Superior	0.268 ± 0.033 | 0.271 (0.223; 0.328)	0.311 ± 0.021 | 0.31 (0.271; 0.353)	0.001
Nasal	0.26 ± 0.046 | 0.257 (0.165; 0.322)	0.304 ± 0.032 | 0.31 (0.219; 0.342)	< 0.001
Inferior	0.268 ± 0.042 | 0.272 (0.175; 0.335)	0.291 ± 0.045 | 0.304 (0.175; 0.339)	0.026
DCP			
Superior	0.262 ± 0.03 | 0.271 (0.197; 0.299)	0.289 ± 0.012 | 0.289 (0.266; 0.312)	0.041

OCT, optical coherence tomography; OCTA, optical coherence tomography angiography; ICP, intermediate capillary plexus; DCP, deep capillary plexus; SVP, superficial vascular plexus

*Represented by mean ± SD and median (min.; max.), respectively

†Generalized estimation equations with normal distribution and identity link function with exchangeable correlation matrix between eyes

### Qualitative findings analysis

**Fig. 1:**
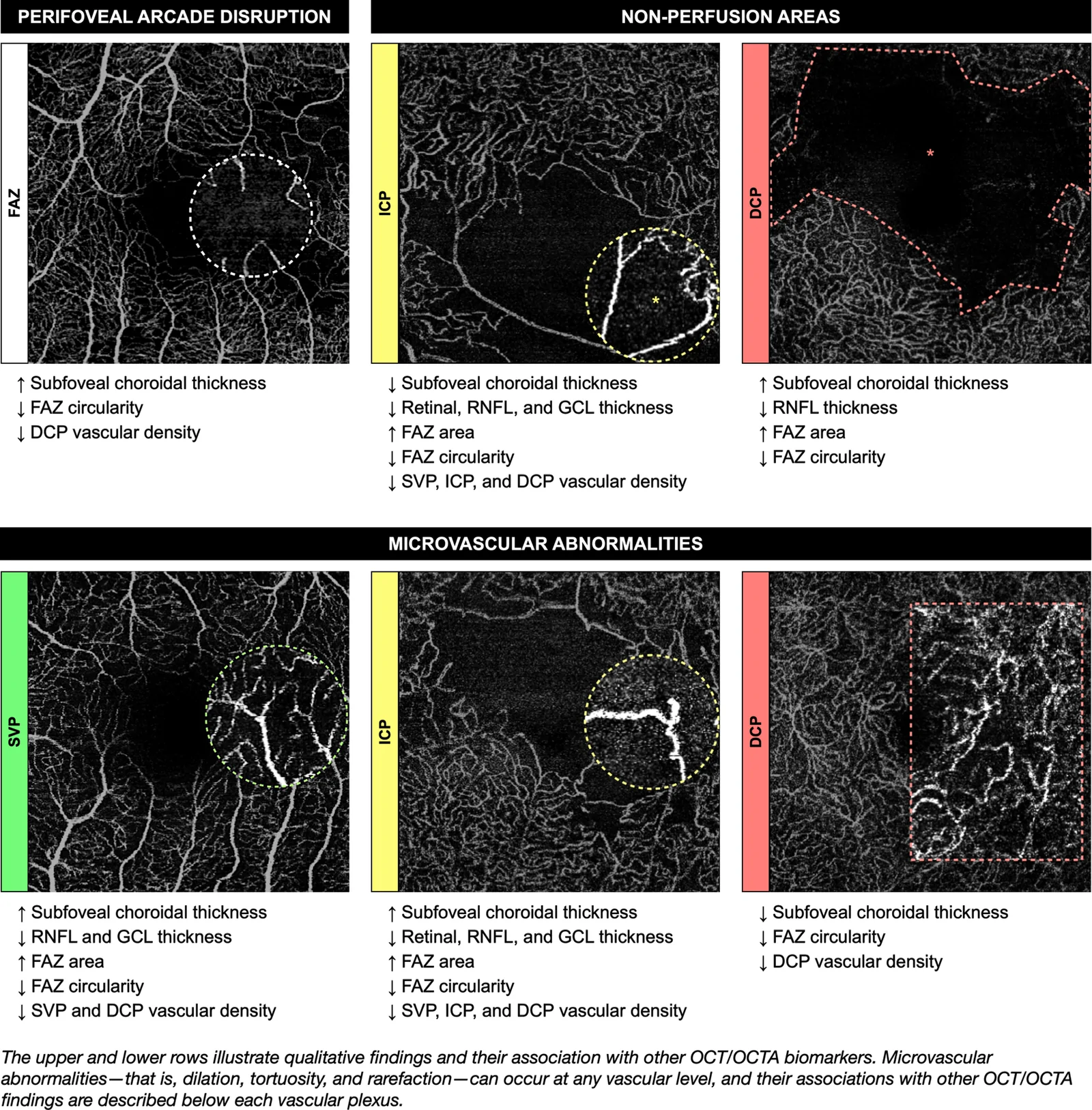
Qualitative findings in optical coherence tomography (OCT) angiography and their correlations with OCT and OCT angiography biomarkers in Behçet’s syndrome-associated retinal vasculitis

Using OCTA, we found perifoveal arcade disruption, NPA, or microvascular abnormalities in 22 eyes (95.7%). These qualitative findings were associated with quantitative changes on OCT, OCTA, and MP (Fig. 1).

### Perifoveal arcade disruption

Disruption of the perifoveal arcade was observed in ten eyes (43.5%). It was associated with an increase in subfoveal choroidal thickness on OCT (*P* < 0.05) and a reduction in the FAZ circularity and the VD of the DCP in the superior sector (*P* < 0.001) on OCTA.

### Non-perfusion areas

In the SVP, NPA were observed in four eyes (17.4%) and were associated (*P* < 0.05) with thinning in the subfoveal choroid, full retina (central, superior, nasal, temporal, and inferior sectors), RNFL (nasal, temporal, and inferior sectors), GCL (superior, nasal, temporal, and inferior sectors), and RNFL + GCL complex (superior, nasal, temporal, and inferior sectors). They were also associated with the enlargement of the FAZ area, SVP VD (global, nasal, and temporal sectors), ICP VD (global, superior, nasal, temporal, and inferior sectors), and DCP VD (global, superior, and temporal sectors).

In the ICP, NPA were observed in six eyes (26.1%) and were associated (*P* < 0.05) with thinning in the subfoveal choroid, RNFL (nasal, temporal, and inferior sectors), GCL (nasal, temporal, and inferior sectors), and RNFL + GCL complex (superior, nasal, temporal, and inferior sectors). They were also associated with the enlargement of the FAZ area and reduction in FAZ circularity, SVP VD (global, nasal, and temporal sectors), ICP VD (global, nasal, and temporal sectors), and DCP VD (superior sector).

In the DCP, NPA were observed in eight eyes (34.8%) and were associated (*P* < 0.05) with greater subfoveal choroid thickness, reduced temporal and inferior RNFL thickness, FAZ area enlargement, and circularity reduction. Among these eyes, six also exhibited NPA in the ICP, and of these, four further presented NPA in the SVP.

### Microvascular abnormalities

In SVP, dilatation, tortuosity, and rarefaction were observed in seven eyes (30.4%). They were significantly (*P* < 0.05) associated with greater choroidal thickness, reduction in RNFL (temporal sector) and GCC thickness (nasal sector), enlargement in the FAZ area and reduction in FAZ circularity, SVP VD (nasal sector), and DCP VD (superior sector). Of the seven eyes with microvascular abnormalities in the SVP, five (71.4%) also presented these in the ICP or DCP.

In ICP, microvascular abnormalities were observed in six eyes (26.1%). They were significantly (*P* < 0.05) associated with greater choroidal thickness, thinning in retinal thickness (global, nasal, temporal, and inferior sectors), RNFL (temporal and inferior sectors), GCL (nasal and inferior sectors) and RNFL + GCL complex (nasal and inferior sectors), enlargement of FAZ area and reduction in FAZ circularity, SVP VD (global, nasal, and superior sectors), ICP VD (nasal, temporal, inferior sectors), and DCP VD (global, superior, and temporal sectors). Among six eyes with microvascular abnormalities in the ICP, three (50%) also presented in the DCP.

In DCP, microvascular abnormalities were observed in 13 eyes (56.5%). They were significantly (*P* < 0.05) associated with a reduced subfoveal choroidal thickness on OCT and FAZ circularity and DCP VD on OCTA (global and inferior sector).

### Structural and functional correlations

**Fig. 2:**
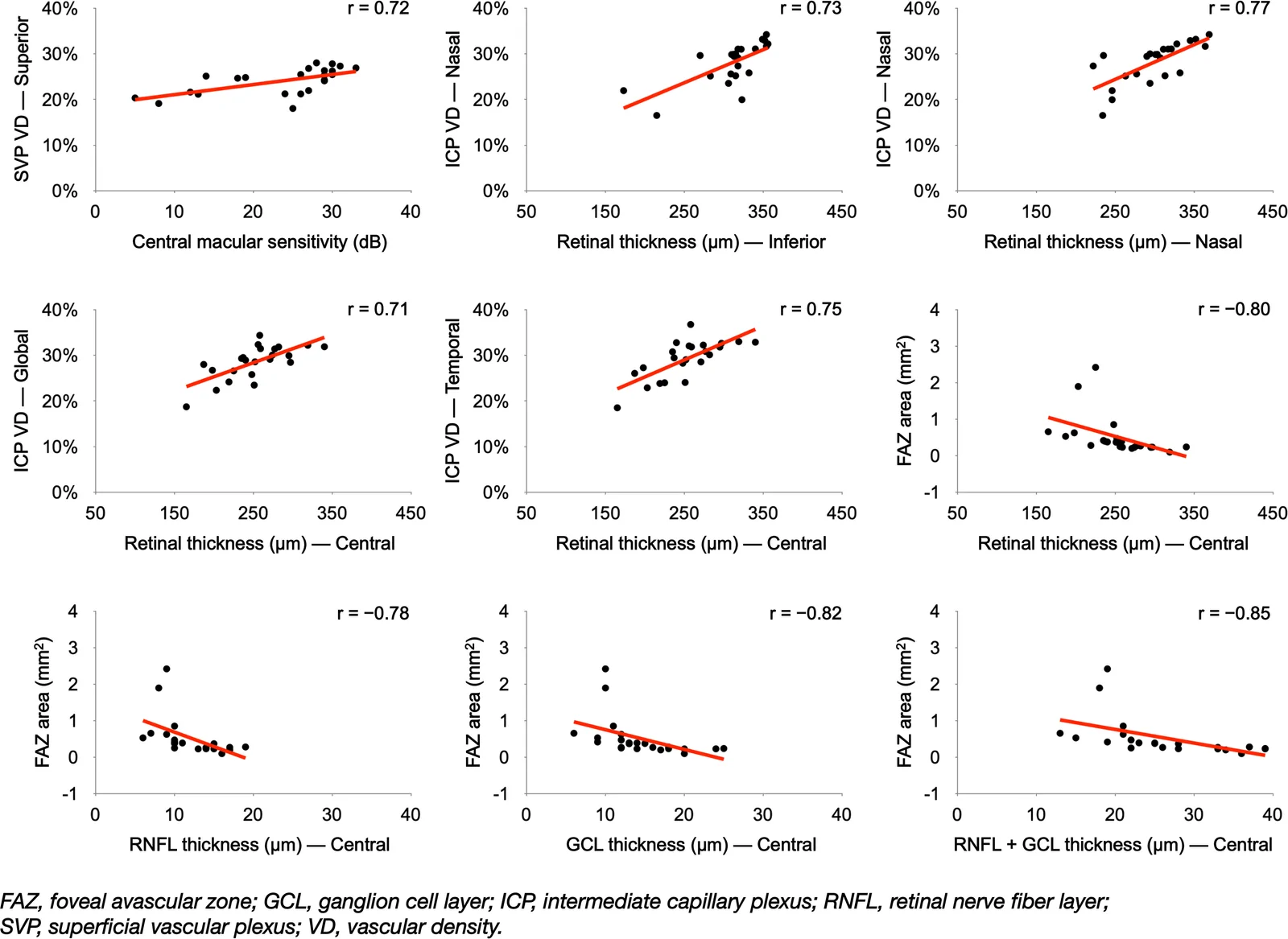
Structure–function correlation between optical coherence tomography (OCT), OCT angiography, and microperimetry variables in patients with Behçet’s syndrome-associated retinal vasculitis

**Fig. 3:**
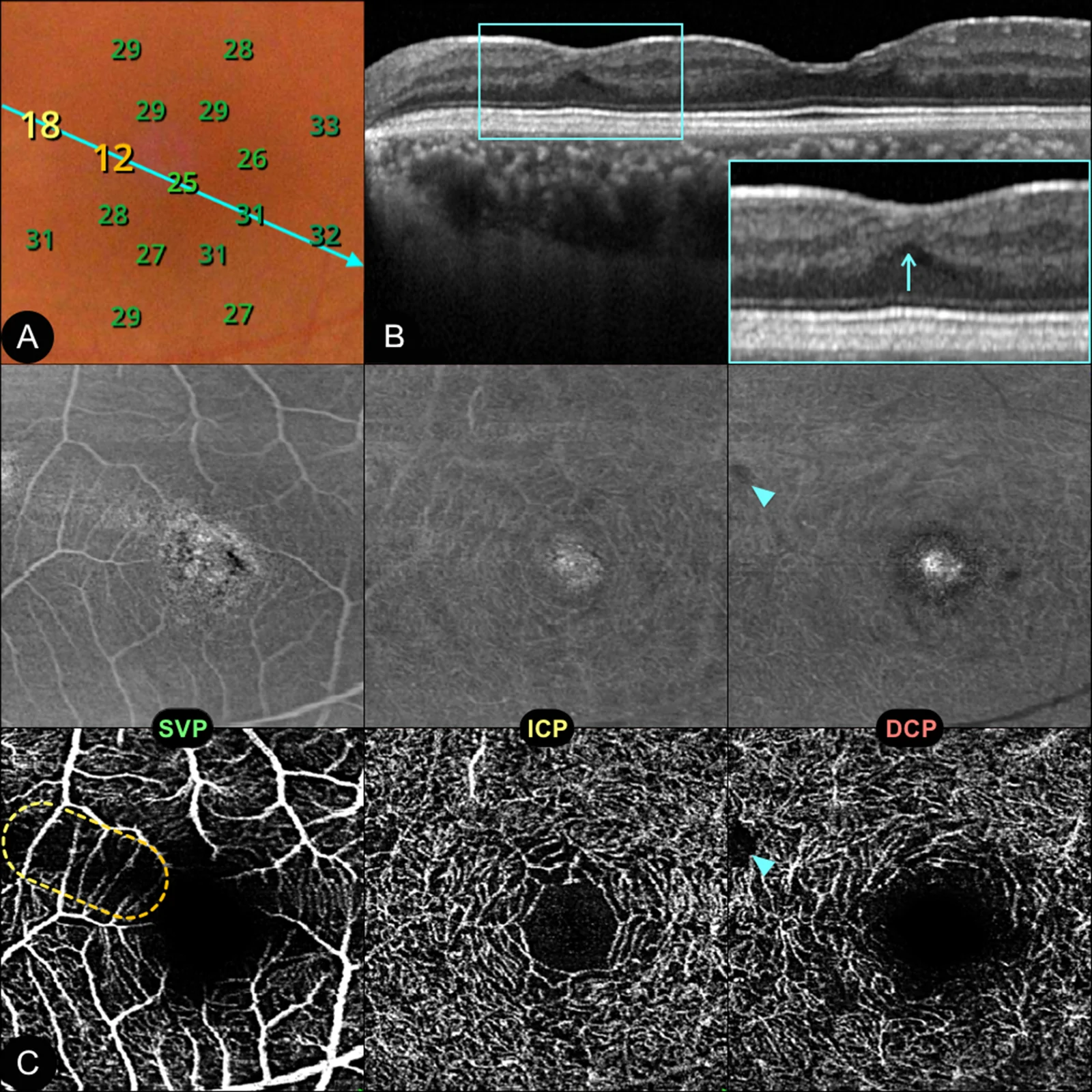
Left eye of a 29-year-old male participant diagnosed with panuveitis associated with Behçet’s syndrome at age 16. (A) Microperimetry showing reduced macular sensitivity, most pronounced in the nasal sector. (B) Optical coherence tomography (OCT) demonstrating inner retinal thinning and elevation in the outer plexiform layer (blue arrow). (**C**) OCT-angiography revealing capillary rarefaction (yellow-orange dashed area) in the superficial vascular plexus and a non-perfusion area in the deep capillary plexus (blue arrowhead)

Using Spearman’s correlation, we identified strong correlations (*r* > 0.7) among data obtained from OCT, OCTA, and microperimetry (Fig. 2). Direct correlations (*P* < 0.05) were evident between superior SVP VD and central MS; nasal ICP VD and inferior and nasal retinal thickness; and central retinal thickness and global and temporal ICP VD. Moreover, inverse correlations (*P* < 0.05) were noted between the FAZ area and thickness in the central retina, RNFL, GCL, and RNFL + GCL complex. A representative case illustrating these structure–function relationships is shown in Fig. 3, where qualitative findings in the SVP, ICP and DCP correspond to reduced retinal thickness and impaired MS.

### Longitudinal evaluation

Seven of our patients were followed up every 4 months for a year. All of them remained with inactive ocular and systemic disease, and there was no change in their BCVA and treatment during this period. No significant alterations were observed in structural or functional parameters on OCTA and microperimetry, respectively, during this period (Table 3).

**Table 3:** OCTA and microperimetry parameters in inactive Behçet’s retinal vasculitis during 12-month follow-up

Variable*	Baseline	12 months	*P*†
OCTA			
FAZ			
Area (mm^2^)	0.705 ± 0.719 | 0.404 (0.097; 2,419)	0.705 ± 0.688 | 0.454 (0.096; 2,252)	0.969
Circularity	0.619 ± 0.234 | 0.725 (0.175; 0.812)	0.625 ± 0.229 | 0.737 (0.195; 0.848)	0.269
Global vascular density (%)			
SVP	0.225 ± 0.027 | 0.225 (0.181; 0.263)	0.233 ± 0.03 | 0.246 (0.176; 0.268)	0.115
ICP	0.28 ± 0.044 | 0.289 (0.187; 0.344)	0.289 ± 0.039 | 0.311 (0.211; 0.328)	0.544
DCP	0.267 ± 0.035 | 0.273 (0.195; 0.309)	0.273 ± 0.039 | 0.281 (0.166; 0.32)	0.613
Microperimetry			
Sensitivity (dB)			
Central macula	25.3 ± 6.1 | 26.5 (12; 31)	25.2 ± 4.6 | 26.5 (16; 30)	0.765
Average	25.8 ± 4.9 | 27.5 (15.1; 31.2)	25.5 ± 4.8 | 26.9 (15.3; 31.2)	0.996

DCP, deep capillary plexus; FAZ, foveal avascular zone; ICP, intermediate capillary plexus; MP, microperimetry; OCTA, optical coherence tomography angiography; SVP, superficial vascular plexus

*Represented by mean ± SD and median (min.; max.), respectively

†Generalized estimation equations with normal distribution and identity link function with autoregressive correlation matrix between moments and eyes; no statistically significant differences were found across the 4-month intervals

## Discussion

This cohort of 14 patients (23 eyes) with inactive BS-associated retinal vasculitis provided several novel observations. that warrant further investigation. Our findings indicated that male and young patients exhibit more prominent structural changes on OCT/OCTA and functional changes on MP. We also found that qualitative biomarkers were correlated with changes in retinal layers and VD. Furthermore, VD was strongly correlated with structural changes on OCT and macular function on MP. Our previous study demonstrated that patients with inactive BS-associated retinal vasculitis had damage mainly in the DCP. We go beyond this and suggest that these qualitative structural abnormalities may indicate a more severe disease affecting the SVP and ICP.

BS affects both sexes. However, the disease tends to be more severe in younger patients and in men compared to woman [19–21]. Our results corroborate these observations, showing more pronounced compromise of retinal vasculature and macular sensitivity in male and young patients. Genetic differences between male and female patients with BS have been described, particularly in the human leucocyte antigen class I region [21]. However, whether these differences influence the disease severity remains unclear. In the present study, sex-related differences were observed in SVP VD and ICP VD, impacting RNFL and GCL thickness and MS. Age-related differences were mainly observed in VD. Compared to our previous study, which revealed differences between BU and non-ocular BS primarily in the VD of DCP with relative sparing of the superior and temporal sectors [10], the present analysis showed that changes may be present in all plexuses and sectors, suggesting a more severe phenotype in male and younger patients. In our cohort, the distribution of sex within each age subgroup was small, preventing a formal analysis comparing younger men versus other subgroups. Nevertheless, younger patients exhibited more pronounced structural and functional impairment despite having shorter disease duration, suggesting that age may be associated with a more aggressive disease phenotype. Given the limited sample size and the fact that the most severely affected eyes were ineligible for formal analysis due to profound fixation loss or advanced tissue disruption, these findings should be interpreted as exploratory.

Interestingly, the qualitative findings were also associated with greater severity in our cohort. NPA and microvascular abnormalities were observed in SVP and ICP, apart from DCP, and were associated with decreased VD in all plexuses. This association has been mentioned with other ischemic vasculopathy in other studies. For example, in retinal venous occlusion, perifoveal arcade disruption was frequently found in cases of macular ischemia [22]. In diabetic retinopathy, NPA were associated with more severe disease and with more structural and functional abnormalities [23, 24]. Moreover, dilated vessels forming loops were associated with NPA and were highly correlated with more severe intraretinal microvascular abnormalities [25].

Using OCT, OCTA, and microperimetry, we identified correlations between structure and function, with several showing strong correlations (*r* > 0.7, Spearman’s correlation coefficient), which should be interpreted with caution given the limited sample size. We speculate that in BU, the direct correlation between the VD of the SVP (superior sector) and central MS could be a possible late involvement of the superior sector of the SVP. A correlation between this plexus and the macular function has been reported in glaucoma [26]. The positive association between inferior and nasal retinal thickness (sectors most affected by BS) with the VD of the ICP (nasal sector) probably occurs because this plexus undergoes alterations earlier than the superficial one (with minimal or no vascular reduction) and later than the DCP (with more significant vascular loss). Another area of exploration is the inverse correlation between the central retinal thickness and FAZ area, which suggests a greater extent of ischemia, thereby resulting in a greater severity of involvement with central atrophy. Additionally, the inverse correlation between the RNFL + GCL complex and FAZ area may occur due to greater severity and extension of ischemia. This results in repercussions in the SVP, which is responsible for irrigating the GCL.

Notably, our data suggest an upstream involvement of retinal microvasculature in BS-associated retinal vasculitis beginning in DCP (particularly in the nasal sector, with the superior and temporal sectors being the last involved), followed by the ICP, and, finally, the SVP. Similarly, in diabetic retinopathy, it has been demonstrated that VD of all plexuses decreases with increasing disease severity [27]. This reduction was observed primarily in the deeper vascular layers (DCP and ICP), with a proportionally more significant decline in SVP across various severity levels of the retinopathy. Therefore, combining information from all three vascular plexuses could also increase the overall ability of OCTA to identify different levels of macular involvement in patients with BS, which may reflect the retinal “ischemic cascade” described in paracentral acute middle maculopathy with progressive vertical ischemia [28, 29]. Conversely, glaucoma preferentially affects superficial macular perfusion so that the density and thickness of the GCL correlate with the visual field [26].

In evaluations conducted every 4 months for a year, no clinical or ocular recurrences were observed, nor were differences detected in clinical, structural, and functional characteristics of OCT, OCTA, and microperimetry. These findings suggest relative stability of structural and functional parameters in patients under clinical remission over the 12-month follow-up. However, the limited sample size precludes definitive conclusions regarding the permanence of these alterations. Indeed, Wassef et al. [30]. described 20 patients (26 eyes) with active Behçet’s posterior uveitis until clinical remission and observed that abnormalities on SCP and ICP were transient, but those in DCP were definitive. This persistence of VD reduction in DCP in BS has been previously described and suggests the obliterative nature of BS-associated retinal vasculitis [31].

This study has several limitations. First, the sample size was modest, reflecting the recruiting challenge of individuals with inactive Behçet’s syndrome–associated retinal vasculitis willing to undergo imaging exams during a 12-month follow-up, and limiting the statistical power, particularly for subgroup analyses. Second, subgroup comparisons were exploratory and hypothesis-generating, and no adjustment for multiple comparisons was performed, increasing the risk of type I error. Third, all imaging analyses were conducted by a single examiner, which may limit reproducibility, although measurements were obtained on two separate occasions to ensure intraobserver consistency. Fourth, inclusion of both eyes may introduce residual inter-eye correlation despite the use of GEE models. Fifth, selection bias may have occurred, as patients with poor fixation or advanced disease were excluded. A higher proportion of excluded eyes due to poor fixation or advanced damage occurred in younger patients, suggesting more severe disease in this subgroup; however, this observation should be interpreted with caution. Additionally, potential confounders, such as disease duration and prior treatment exposure, were not fully controlled. Finally, OCTA analysis was limited to a 3 × 3 mm macular area, which may not capture more peripheral microvascular changes [32].

In conclusion, this study provides exploratory insights into retinal microvascular and functional alterations in BS–associated retinal vasculitis. Our findings suggest that male sex and younger age may be associated with patterns indicative of greater macular structural and functional involvement. Qualitative OCTA biomarkers were associated with quantitative changes on OCT, OCTA, and microperimetry, supporting their potential role in characterizing disease-related microvascular damage. Involvement of the SVP and ICP may reflect more extensive retinal microvascular impairment. Future studies with larger cohorts are required to validate these observations.

## Data Availability

The datasets generated and analyzed during the current study are available from the corresponding author upon reasonable request.

## Declarations

### Authors information

This study was conducted as part of the doctoral research project of B.F.A.F. in Ophthalmology at the Faculdade de Medicina, Universidade de São Paulo.

## Abbreviations

BCVA: Best–corrected visual acuity
BR: BDCAF–Brazilian–adapted Behçet’s Disease Current Activity Form
BS: Behçet’s syndrome
BU: Behçet’s uveitis
DCP: Deep capillary plexus
FA: Fluorescein angiography
FAZ: Foveal avascular zone
GCL: Ganglion cell layer
ICP: Intermediate capillary plexus
MP: Microperimetry
MS: Macular sensitivity
NPA: Non–perfusion areas
OCT: Optical coherence tomography
OCTA: Optical coherence tomography angiography
RNFL: Retinal nerve fiber layer
SVP: Superficial vascular plexus
VD: Vascular density
